# Global burden of cancer attributable to high BMI (1990–2031): a multidimensional analysis based on GBD and Mendelian randomization

**DOI:** 10.3389/fnut.2025.1618799

**Published:** 2025-08-05

**Authors:** Xianglin Zhu, Yushuai Mi, Lang Wang, Hao Liang, Jie Zhang, Shijun Zhao, Cheng Zhao, Yinlu Ding

**Affiliations:** ^1^Department of Gastrointestinal Surgery, The Second Hospital of Shandong University, Jinan, China; ^2^The Second Clinical Medical College, Cheeloo College of Medicine, Shandong University, Jinan, China

**Keywords:** global burden of disease, Mendelian randomization, high body mass index, cancer, mortality, disability-adjusted life years

## Abstract

**Objective:**

Obesity-related health burdens have emerged as particularly intractable public health issues on a global scale. This study aims to analyze the association between body mass index (BMI) and 12 types of cancer, examine the regional, gender, and age disparities in cancer burden attributable to high BMI, and project the disease burden trends over the next decade based on available data.

**Methods:**

Data for this study were sourced from the Integrative Epidemiology Unit (IEU) Open Genome-Wide Association Study (GWAS) Project and the 2021 Global Burden of Disease (GBD) database. Using Mendelian randomization (MR), we investigated the association between BMI and 12 cancer types. We also collected and analyzed epidemiological data on cancers attributable to high BMI, calculated the estimated annual percentage change (EAPC) across 21 regions, and examined disparities in mortality and disability-adjusted life years (DALYs) by age, sex, and cancer type. Finally, we used the autoregressive integrated moving average (ARIMA) model to predict trends in various cancers attributable to high BMI over the next 10 years.

**Results:**

In 2021, high BMI accounted for 356,738 cancer deaths worldwide and 8,894,525 DALYs, representing an increase of 160% in deaths and 151% in DALYs compared to 1990 (which recorded 137,353 deaths and 3,549,049 DALYs). Among the cancers attributable to high BMI, colon and rectal cancer accounted for the highest disease burden, while thyroid cancer accounted for the lowest proportion of disease burden. Gender-stratified analysis revealed a notably higher disease burden among women compared to men. An age-specific assessment revealed a disproportionately higher disease burden in the 50–79 age cohort. Additionally, both the age-standardized mortality rate (ASMR) and age-standardized disability rate (ASDR) showed positive correlations with the Socio-demographic Index (SDI). Finally, projections from the ARIMA model indicate that over the next decade, the ASMR for most cancers attributable to high-BMI will remain stable or increase, except for colon, rectal, and uterine cancers. The MR analysis indicated a causal relationship between BMI and 11 cancer types (colon and rectal cancer, liver cancer, gallbladder and biliary tract cancer, pancreatic cancer, breast cancer, uterine cancer, ovarian cancer, kidney cancer, lymphoma, multiple myeloma, and leukemia), while no causal association was found between BMI and thyroid cancer.

**Conclusion:**

Mendelian randomization analysis indicated a notable association between elevated BMI and an increased risk of 11 cancer types. Over the past three decades, the cancer burden attributable to high BMI has demonstrated a marked increasing trend, with notable variations observed across geographic regions, gender groups, and age categories regarding predominant cancer types. These findings underscore the need to develop targeted prevention strategies and health promotion interventions that are tailored to specific demographic and regional profiles.

## Introduction

According to the World Health Organization (WHO), one in eight people worldwide lived with obesity in 2022, with the global prevalence more than doubling between 1990 and 2022 (1). This trend demonstrates that the rising prevalence of high BMI worldwide has emerged as a critical public health challenge. In addition to the well-established associations with cardiovascular diseases, kidney diseases, and diabetes (2–4), growing clinical evidence indicates a positive correlation between high body mass index (BMI) and cancers such as gallbladder and biliary tract cancer, pancreatic cancer, and leukemia, among others (5–8). This suggests that BMI is an important factor in the onset and progression of cancer.

The impact of high-BMI-attributable diseases extends beyond impairing patients’ physical and mental health as well as daily functioning. It also exerts profound effects on families and overall social development due to rising healthcare expenditures, premature mortality, and productivity losses caused by work absenteeism (9, 10). According to WHO research findings, if current prevention and control measures remain unchanged, the global economic losses attributable to overweight and obesity are projected to escalate to USD 3 trillion annually by 2030 (1).

Several previous studies have investigated the cancer burden attributable to high BMI; however, these analyses were based on the 2019 Global Burden of Disease (GBD) database (11, 12). In the latest 2021 GBD database, the number of cancer types attributable to high BMI has changed from the previously reported 13 to 12, with esophageal cancer no longer included. This modification has consequently altered the proportional contributions among the remaining obesity-related cancers. Although there are studies that utilized the 2021 database, the first focused on decomposition analysis to assess the key contributors to the increasing cancer burden attributable to overweight (13), while the second adopted a holistic approach to examine age, sex, and SDI-regional disparities in BMI-associated cancer burden without detailed stratification by specific cancer types (14). Meanwhile, previous studies have not analyzed the proportional contribution of different cancer types across various age groups. Therefore, this study utilizes the latest 2021 GBD database to investigate the burden of different cancer types attributable to high BMI, stratified by geographic region, economic region, sex, and age group. Additionally, it projects the trends in cancer burden over the next decade. Furthermore, when studying cancers attributable to BMI, the findings may be influenced by various confounding factors, such as lifestyle variables (physical activity and smoking), which could obscure the establishment of definitive causal inferences. To address these limitations and rigorously evaluate the causal relationship between BMI and cancer risk, we employed an innovative Mendelian randomization approach to minimize confounding bias in our study, thereby strengthening the validity of our findings (15).

## Methods

### Overview

The data used in this study were sourced from the 2021 GBD database^1^ and the IEU Open Genome-Wide Association Study (GWAS) project^2^ (16–18). The GBD database encompasses 371 diseases and injuries, along with 88 risk factors, across 204 countries, spanning 21 geographical regions and five economic zones worldwide (19–22). The GBD database is an internationally authoritative health database developed and maintained by the Institute for Health Metrics and Evaluation (IHME) at the University of Washington. It is openly accessible through the Global Health Data Exchange (GHDx) platform (23). The database’s groundbreaking innovation lies in its application of advanced computational models, including Bayesian mixed-effects meta-regression (DisMod-MR 2.1) and spatiotemporal Gaussian process regression (ST-GPR), to standardize and calibrate the raw data (23, 24). This methodology effectively addresses critical challenges in data comparability across sources and imputes missing values, thereby generating temporally and spatially comparable estimates of key metrics such as incidence rates, mortality, and disability-adjusted life years (DALYs). The GWAS summary statistics for exposure/outcome factors used in this study were sourced from the MRC Integrative Epidemiology Unit (IEU) Open GWAS Project (IEU Open GWAS) (25, 26). This project (see text footnote 2) constitutes a large-scale, open-access repository of rigorously standardized GWAS summary statistics.

### SDI

The Sociodemographic Index (SDI) is a composite metric that comprehensively evaluates the socioeconomic status of regions or nations by integrating three core dimensions: total fertility rate, educational attainment, and per capita income (27). The comprehensive evaluation adopts a 0–1 standardized scoring system, where higher development levels correspond to scores closer to 1. In the GBD 2021 study, both the 21 regions and 204 countries were categorized into five distinct development tiers based on the aforementioned scoring system: high SDI, upper-middle SDI, middle SDI, lower-middle SDI, and low SDI (28). By incorporating the SDI, we systematically analyzed temporal trends in high-BMI-attributable cancer mortality and DALYs across 21 geographic regions from 1990 to 2021, with a particular focus on their association with economic development levels.

### ARIMA

The autoregressive integrated moving average (ARIMA) model is a classical time-series forecasting method that integrates three key components: autoregression (AR), differencing (I), and moving average (MA) (29, 30). It is particularly effective in handling non-stationary time-series data for accurate predictions. It is commonly denoted as ARIMA (p,d,q), where p, d, and q represent the autoregressive order, differencing for stationarity, and moving average order, respectively (31, 32). Three steps, namely, (a) a stationarity test, (b) model identification and order determination, and (c) model diagnosis, were used to fit the best ARIMA model (33). Using the ARIMA model, we projected trends (2022–2031) in age-standardized mortality rate (ASMR) for twelve high-BMI-attributable cancer types.

### Mendelian randomization analysis

The two-sample MR analysis utilized publicly available large-scale GWAS datasets, employing genetic variants associated with the exposure as instrumental variables (IVs) (34). This approach is designed to establish causal links between exposures (risk factors) and outcomes (diseases), circumventing the inherent limitations of observational studies. This MR study adheres to the three key assumptions: (1) genetic instruments strongly predict exposure (relevance), (2) instruments are unconfounded (independence), and (3) instruments affect the outcome only via exposure (no horizontal pleiotropy) (35, 36). By employing MR, we effectively mitigated the confounding effects of variables such as smoking and alcohol consumption in examining the association between BMI and cancer, thereby enabling causal inference between the exposure (BMI) and outcomes (twelve distinct cancer types).

### Statistical analysis

This study utilized data from the GBD 2021 database to obtain mortality and DALYs estimates for twelve high BMI-attributable cancers (colon and rectal cancer, liver cancer, gallbladder and biliary tract cancer, pancreatic cancer, breast cancer, uterine cancer, ovarian cancer, kidney cancer, thyroid cancer, Non-Hodgkin lymphoma, multiple myeloma, and leukemia) across 1990–2021. The data were comprehensively stratified by seven super-regions, 21 geographic regions, and five SDI levels, with additional breakdowns by sex and age groups to enable detailed epidemiological analyses. We used ASMR and age-standardized disability rate (ASDR) to eliminate biases arising from variations in population age structures across different time periods and geographic regions (37, 38). We employed the estimated annual percentage change (EAPC) to characterize the trends in ASMR and ASDR from 1990 to 2021. If the EAPC and its uncertainty intervals are less than 0, the corresponding ASR is considered to show a downward trend. Conversely, if the EAPC and its corresponding confidence interval are greater than 0, the corresponding ASR is considered to show an upward trend.


y=α+βx+ε

EAPC=100×(exp(β)−1).

Among them, y=ln(age−standardized rate ), x = year, ε =Residual.

This study also employed a two-sample MR approach to examine causal relationships between BMI and 12 different types of cancer. In the initial step, we identified BMI-associated SNPs (*p* < 5 × 10^−8^) from the IEU Open GWAS project, applying LD clumping thresholds (r^2^ < 0.001, window size = 10,000 kb) to ensure independence. These instrumental variables were then applied to the respective outcome datasets. All included SNPs in our study exhibited F-statistics greater than 10, indicating that they were strong instrumental variables. Detailed information on the instrumental variables (IVs) identified for relevant exposures and outcomes is presented in Supplementary Table S5.

All computations were performed using R Studio version 4.4.1.

## Results

### Temporal trends in mortality rates and DALYs of cancers attributable to high BMI

**Figure 1:**
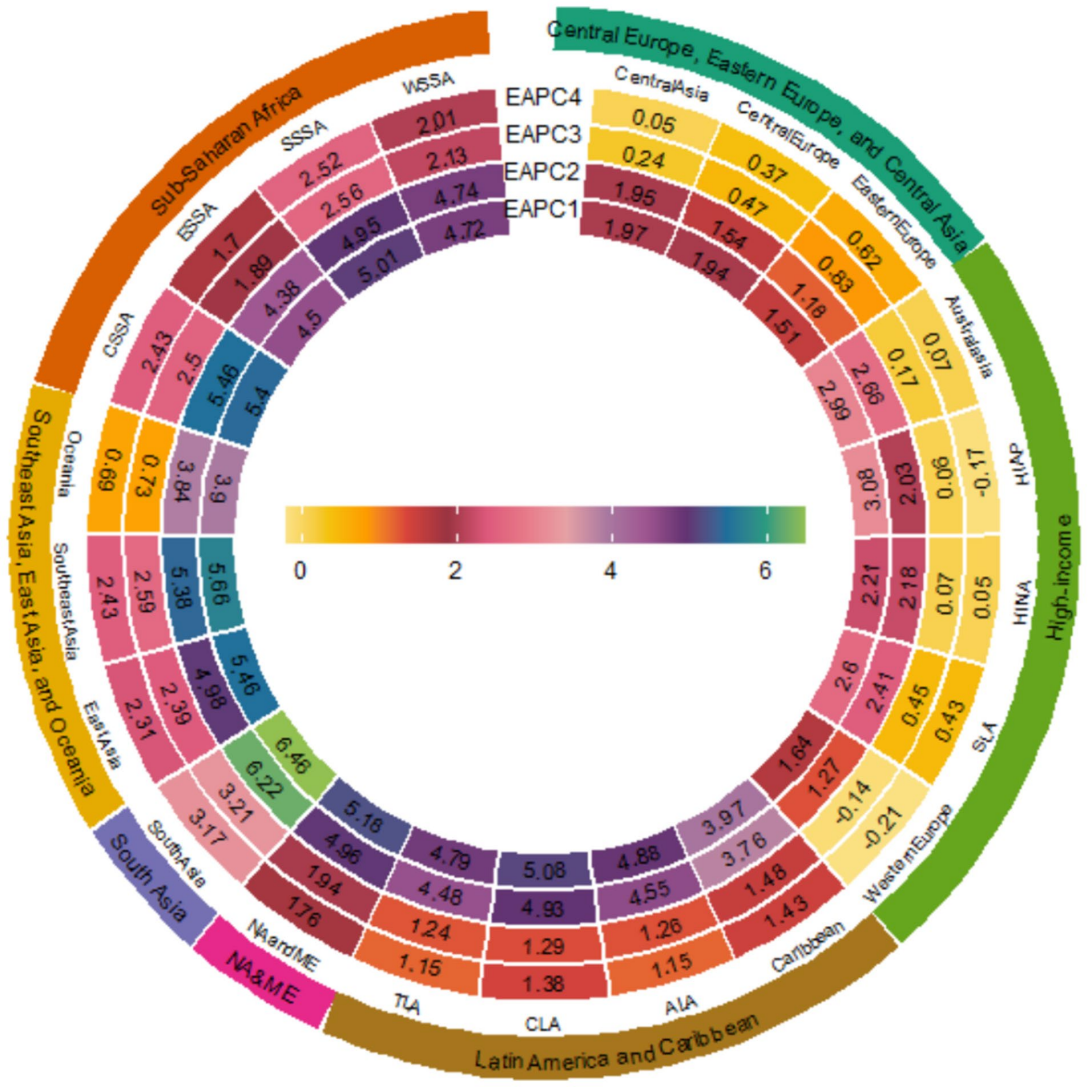
EAPCs of the 21 GBD regions from 1990 to 2021. (EAPC1 = EAPC_Mortality_, EAPC2 = EAPC_DALYs_, EAPC3 = EAPC_ASMR_, EAPC4 = EAPC_ASDR_) (SLA is Southern Latin America; SSSA is Southern sub-Saharan Africa; CSSA is Central sub-Saharan Africa; HINA is High-income North America; NA and ME are North Africa and Middle East; TLA is Tropical Latin America; HIAP is High-income Asia Pacific; ESSA is Eastern sub-Saharan Africa; ALA is Andean Latin America; CLA is Central Latin America; WSSA is Western sub-Saharan Africa).

According to Supplementary Tables S1, S2, in 2021, the global deaths and DALYs number of cancers attributable to high BMI reached 356,738 (95% UI: 146,116 to 581,012) and 8,894,525 (95% UI: 3,751,953 to 14,385,271) respectively, showing a notable increase compared to 1990 [Deaths: 137,353 (95%UI: 57,450 to 225,297); DALYs: 3,549,049 (95%UI: 1,548,429 to 5,731,481)]. The ASMR and ASDR also increased from 3.66 (95% UI: 1.51 to 6.03) and 87.53 (95% UI: 37.43 to 141.83) in 1990 to 4.18 (95% UI: 1.71 to 6.80) and 102.17 (95% UI: 43.24 to 165.02) in 2021, respectively (EAPC_ASMR_ = 0.35, EAPC_ASDR_ = 0.42). From 1990 to 2021, among the 12 cancer types attributable to high BMI, pancreatic cancer and liver cancer showed the most notable increases in ASMR and ASDR (with the largest EAPC values). Conversely, decreasing trends were observed for gallbladder and biliary tract cancer along with leukemia (Supplementary Tables S1, S2). From a regional perspective across 21 geographical areas, all regions experienced consistent increases in cancer burdens attributable to high BMI, specifically manifested as mortality and DALYs, throughout the 32 years from 1990 to 2021 (Figure 1). The most dramatic escalation occurred in South Asia (EAPC for mortality = 6.46; EAPC for DALY = 6.22), while Eastern Europe showed relatively modest increases (EAPC for mortality = 1.51; EAPC for DALYs = 1.18) (Figure 1). South Asia demonstrated the most pronounced increases in both ASMR and ASDR, with EAPC of 3.21 and 3.17, respectively (Figure 1).

### Burden of cancer subtypes attributable to high BMI across 21 regions in 2021

**Figure 2:**
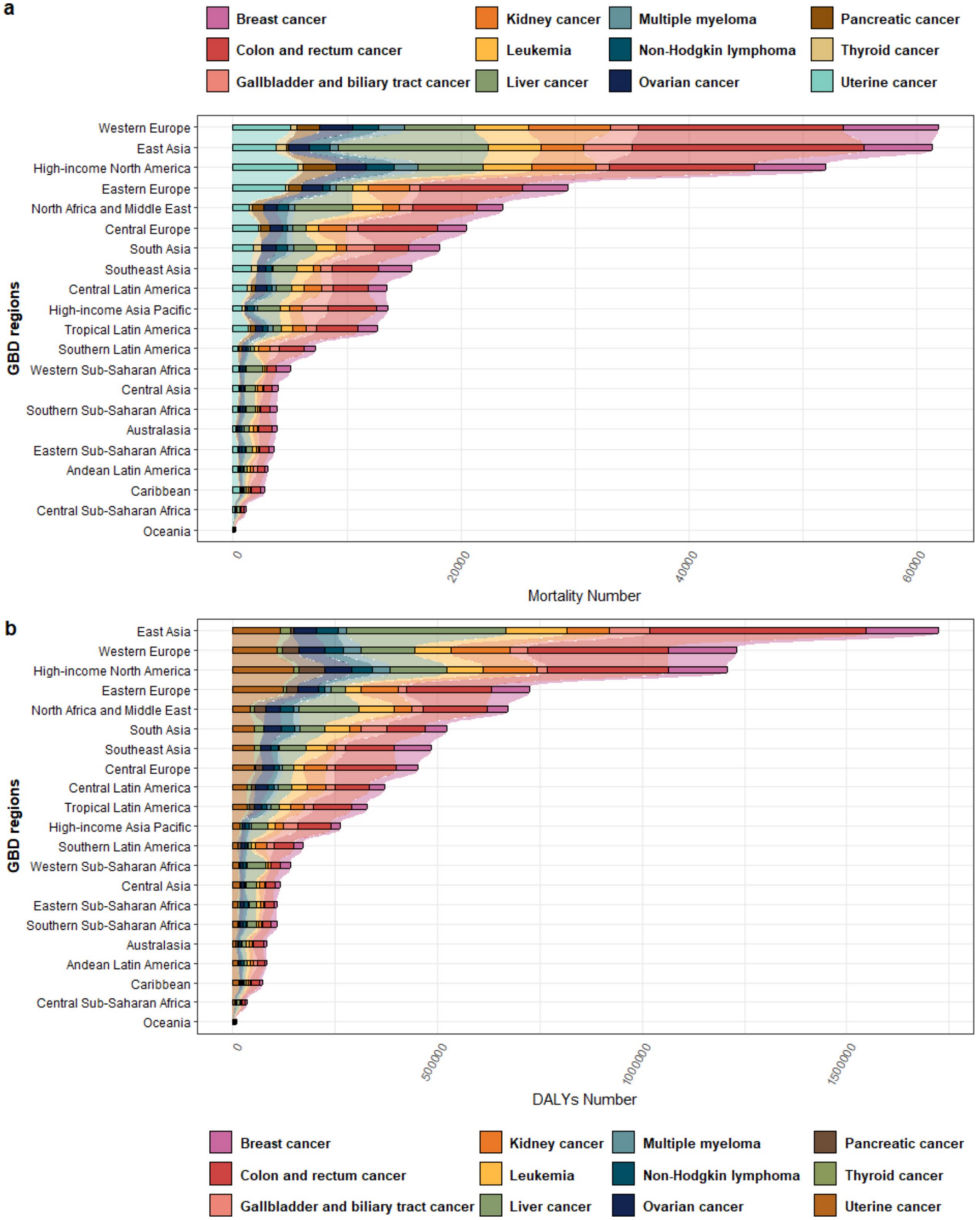
Proportional distribution of different cancer types attributable to high BMI across various regions in 2021: **(a)** Mortality; **(b)** DALYs.

In 2021, Western Europe recorded the highest mortality burden (61,942 deaths). Colorectal cancer accounted for the largest proportion of deaths, followed by breast cancer and kidney cancer, whereas thyroid cancer contributed the smallest proportion (Supplementary Table S1, Figure 2a). In terms of DALYs, East Asia bore the heaviest burden (1,724,414), with colon and rectal cancer still contributing the most, while liver cancer rose to the second highest contributor, followed by breast cancer in third place (Supplementary Table S2, Figure 2b). In terms of both mortality and DALY burden, Oceania had the smallest burden.

### Sex and age disparities in cancer burden attributable to high BMI

**Figure 3:**
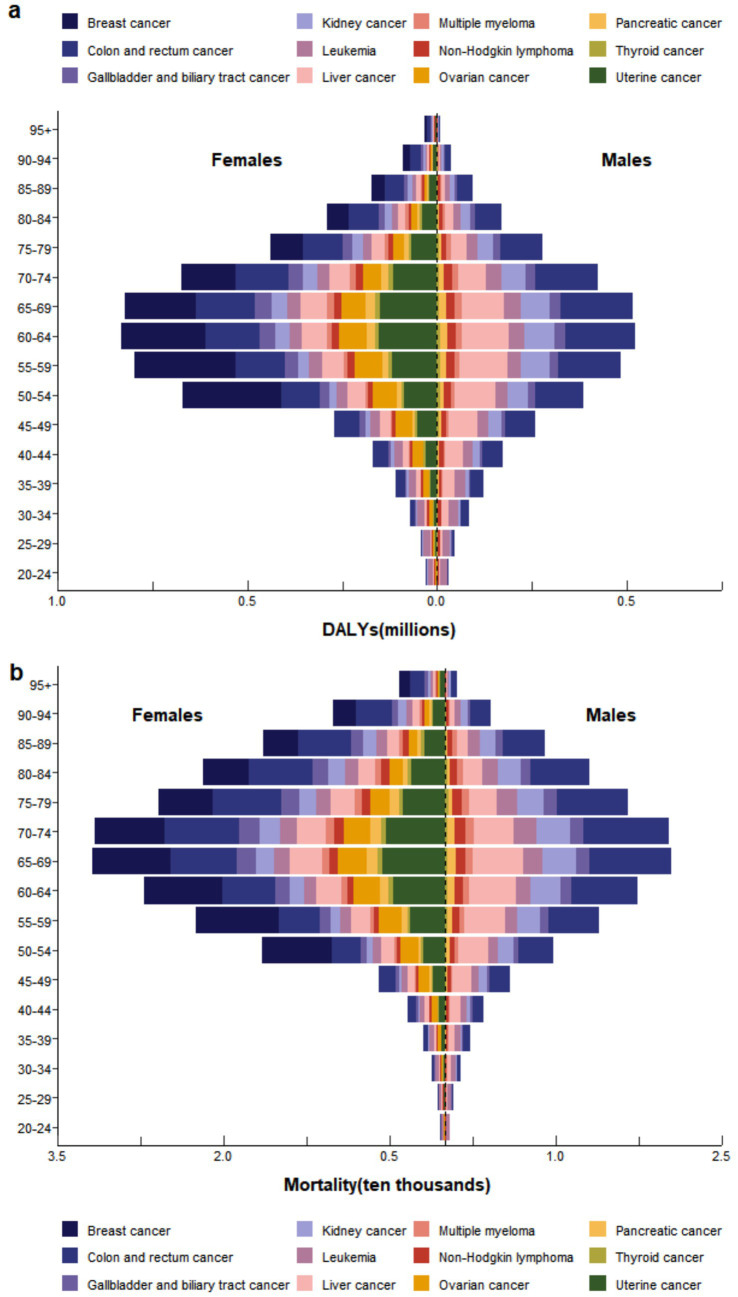
In 2021, the burden of **(a)** DALYs and **(b)** Mortality attributable to high BMI by cancer type, stratified by sex and age group.

In 2021, the number of female deaths and DALYs was nearly twice that of males (Supplementary Table S1, Figure 3a). The primary contributors to cancer-related mortality and DALY burden in women were colon and rectal cancer, breast cancer, and uterine cancer, while for men, the leading contributors were colon and rectal cancer, liver cancer, and kidney cancer (Figure 3). For both males and females, thyroid cancer contributed the least to the overall disease burden. In terms of age distribution, the disease burden was predominantly concentrated in the 50–79 age group. The number of cancer deaths and DALY burden attributable to high BMI began to increase from the 20–24 age group, peaked in the 65–69 and 60–64 age ranges respectively, and then gradually declined (Figure 3). The proportional contributions of different cancer types varied across these peak age ranges. For mortality burden, colon and rectal cancer, liver cancer, and breast cancer were the top three contributors. For DALY burden, the leading contributors were colon and rectal cancer, breast cancer, and liver cancer.

### Regional economic differences in time trends of proportional contributions by cancer type

**Figure 4:**
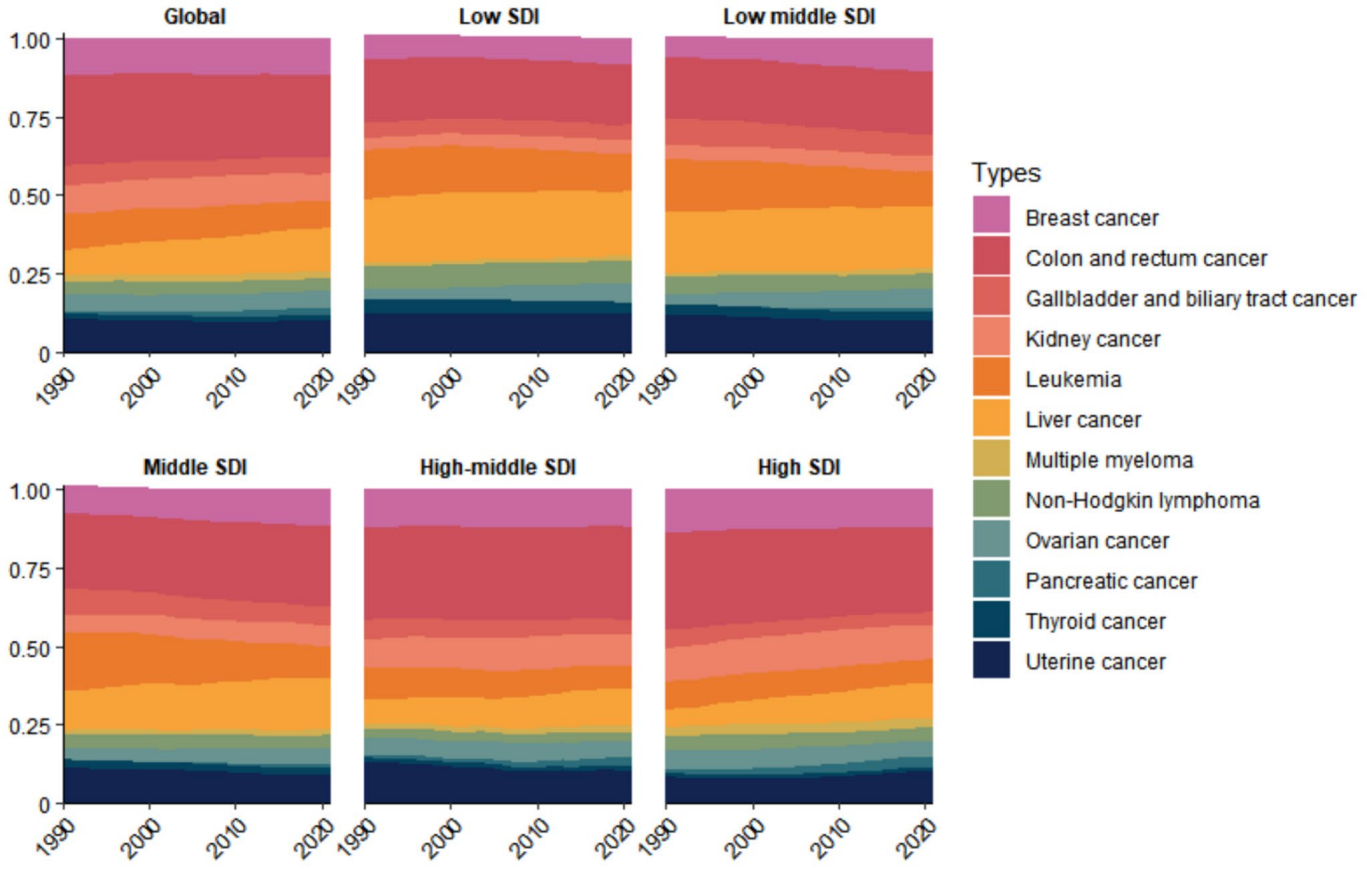
Temporal trends in the proportional distribution of cancer types attributable to high BMI across global and different socioeconomic regions, 1990–2021 (DALY).

For DALY burden, colon and rectal cancer were the primary contributors in Global, High SDI, High-middle SDI, Middle SDI, and Low-middle SDI regions, whereas liver cancer was the leading contributor in Low SDI regions (Figure 4). For mortality burden, colon and rectal cancer was the predominant contributor in all regions except Low-middle SDI areas, where liver cancer represented the primary cause (Supplementary Figure S1). From 1990 to 2021, the proportional contribution of liver cancer to the disease burden gradually increased in most economic regions, while that of leukemia progressively decreased. However, the total burden caused by various cancer types showed a year-by-year increase. Notably, regions with higher economic development levels exhibited heavier mortality and DALY burdens (Supplementary Figures S2, S3). The ASMR and ASDR of cancers attributable to high BMI were generally positively correlated with SDI. That is, ASMR and ASDR gradually increased with rising SDI levels (Supplementary Figure S4).

### Projection of ASMR over the next decade

**Figure 5:**
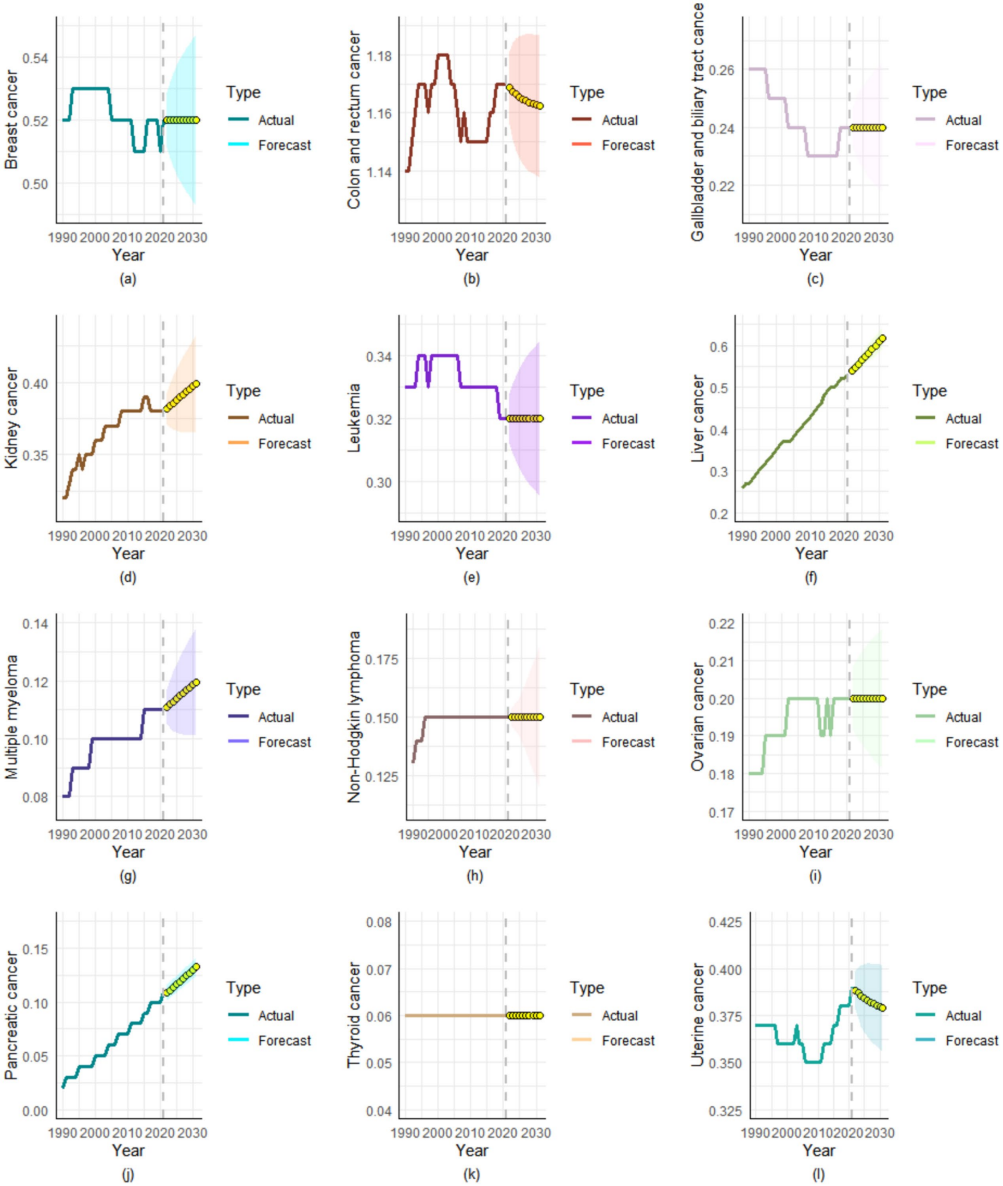
Projected ASMR trends for high BMI-attributable cancer types over the next decade. **(a)** Breast cancer, **(b)** Colon and rectum cancer, **(c)** Gallbladder and biliary tract cancer, **(d)** Kidney cancer, **(e)** Leukemia, **(f)** Liver cancer, **(g)** Multiple myeloma, **(h)** Non-Hodgkin lymphoma, **(i)** Ovarian cancer, **(j)** Pancreatic cancer, **(k)** Thyroid cancer, **(l)** Uterine cancer.

Through ARIMA modeling, we found that over the next decade (2022–2031), the ASMR for most cancers will either increase or remain stable, with only colon and rectal cancer and uterine cancer showing decreasing ASMR trends (Figure 5). By 2031, the ASMR of the following four types of cancers is projected to reach new heights: kidney cancer (0.3994 per 100,000), liver cancer (0.6171 per 100,000), multiple myeloma (0.1197 per 100,000), and pancreatic cancer (0.1327 per 100,000) (Supplementary Figure S3).

### Causal association between BMI and cancer

**Figure 6:**
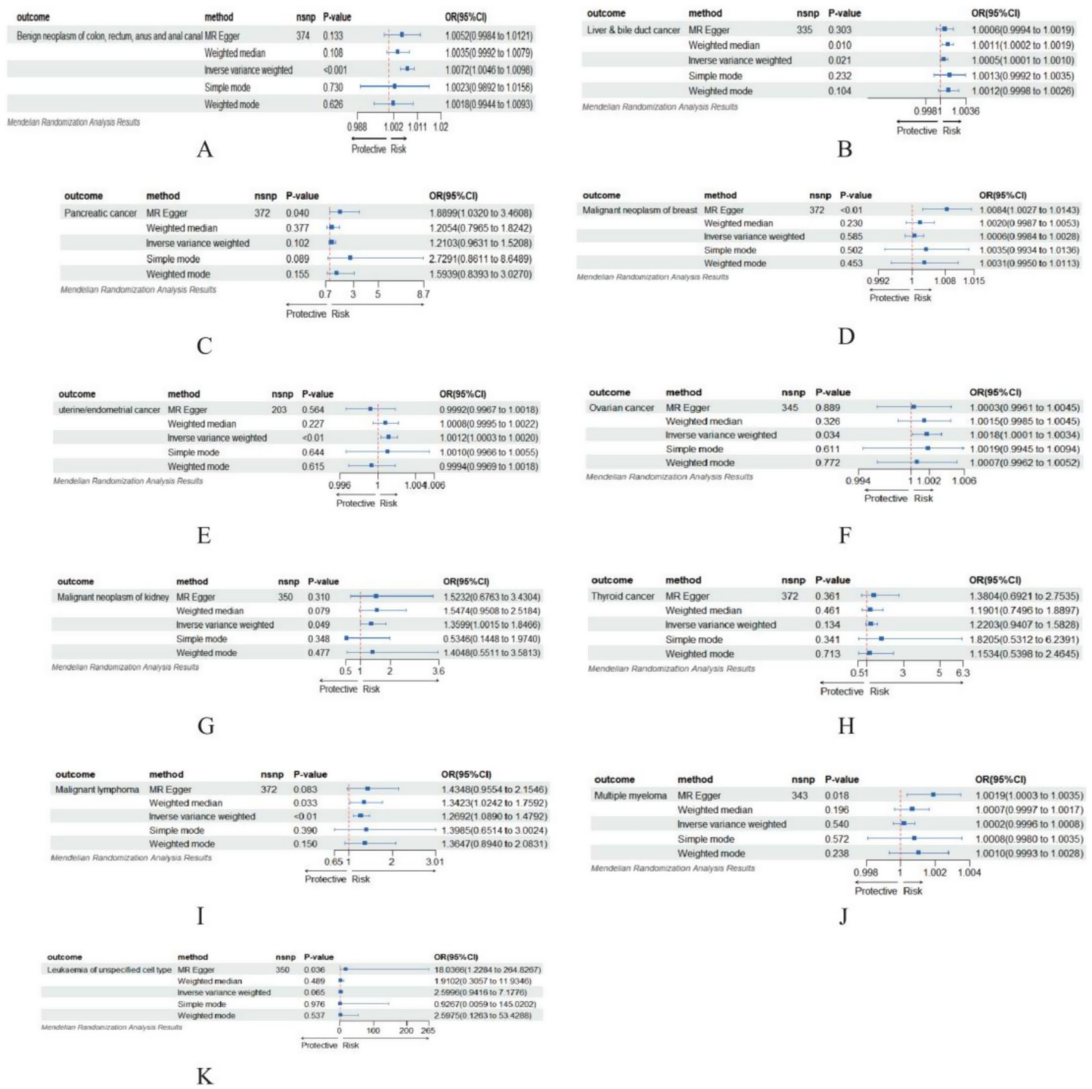
MR-Egger, weighted median, IVW, simple median, and weighted methods were performed to analyze the effect of BMI on different cancers. **(A)** Benign neoplasm of colon, rectum, anus and anal canal; **(B)** Liver & bile duct cancer; **(C)** Pancreatic cancer; **(D)** Malignant neoplasm of breast; **(E)** Uterine/endometrial cancer; **(F)** Ovarian cancer; **(G)** Malignant neoplasm of kidney; **(H)** Thyroid cancer; **(I)** Malignant lymphoma; **(J)** Multiple myeloma; **(K)** Leukemia of unspecified cell type.

Our analysis identified 11 causal relationships between BMI and the 12 investigated cancer types. Our Mendelian randomization analysis demonstrated notable positive associations between elevated BMI and multiple cancer outcomes, including benign neoplasm of colon, rectum, anus and anal canal (OR = 1.0072, 95% CI: 1.0046–1.0098, *p* = 4.77 × 10^−8^), liver and bile duct cancer (OR = 1.0005, 95% CI: 1.0001–1.0010, *p* = 0.021), pancreatic cancer as estimated by MR-Egger regression (OR = 1.8899, 95% CI: 1.0320–3.4608, *p* = 0.040), malignant neoplasm of breast (MR-Egger OR = 1.0084, 95% CI: 1.0027–1.0143, *p* = 0.004), uterine/endometrial cancer (OR = 1.0012, 95% CI: 1.0003–1.0020, *p* = 0.008), ovarian cancer (OR = 1.0018, 95% CI: 1.0001–1.0034, *p* = 0.034), malignant neoplasm of kidney (OR = 1.3600, 95% CI: 1.0015–1.8466, *p* = 0.049), malignant Lymphoma (OR = 1.2692, 95% CI: 1.0890–1.4792, *p* = 0.002), multiple myeloma (MR-Egger OR = 1.0019, 95% CI: 1.0003–1.0035, *p* = 0.018), and leukemia of unspecified cell type (MR-Egger OR = 18.0366, 95% CI: 1.2284–264.8267, *p* = 0.036), collectively indicating BMI’s role as an important risk factor for various malignancies (see Figure 6). However, we found no evidence linking BMI to thyroid cancer (all *p* > 0.05).

## Discussion

To the best of our knowledge, this represents the most comprehensive study to date, integrating GWAS and GBD databases to investigate both the causal relationships between BMI and various cancers and the disease burden attributable to high BMI. This study, utilizing the 2021 GBD database, found that the global cancer burden attributable to high BMI showed a notable upward trend between 1990 and 2021. This trend exhibited notable heterogeneity across geographic regions, economic regions, cancer types, sex, and age groups. We also found that the burden level was closely associated with socioeconomic development, with high SDI regions exhibiting a greater overall burden than low-SDI regions. Finally, we used the ARIMA model to predict the trends of various cancers attributable to high BMI over the next decade.

Firstly, our Mendelian randomization analysis confirmed causal associations between BMI and multiple malignancies (colon and rectal cancer, liver cancer, gallbladder and biliary tract cancer, pancreatic cancer, breast cancer, uterine cancer, ovarian cancer, kidney cancer, Lymphoma, multiple myeloma, and leukemia), while demonstrating no notable causal relationship with thyroid cancer. This finding appears inconsistent with the results reported in the GBD database. Integrating the characteristics of Mendelian randomization and the GBD database yields two key conclusions: First, the oncogenesis of thyroid cancer involves multifactorial determinants. If BMI-associated SNPs influence thyroid cancer development through non-BMI-mediated pathways (modulating inflammatory responses or hormonal regulation) (39). This pleiotropic effect may attenuate the estimated causal association between BMI and cancer risk. Secondly, while the GBD incorporates detailed thyroid cancer subtypes (papillary carcinoma, follicular carcinoma, and so on) in its statistical compilation, MR analyses typically treat thyroid cancer as a unitary endpoint. This methodological discrepancy may account for the null association observed between BMI and thyroid cancer.

In 2021, high BMI contributed to 356,738 global cancer deaths and 8,894,525 DALYs, marking a notable increase compared to 1990. Meanwhile, the continued rise in ASMR and ASDR (with EAPCs of 0.35 and 0.42, respectively) indicates that the cancer burden attributable to high BMI continues to worsen, even after accounting for population growth and aging. Obesity, as a risk factor for cancer, promotes tumorigenesis and progression through multiple mechanisms, including chronic inflammation (40, 41), insulin resistance (42), and disruption of gut microbiota (43). Over the past 32 years, pancreatic cancer and liver cancer demonstrated the most pronounced increases in ASR among the 12 high BMI-attributable cancers. This phenomenon may be attributed to the increasingly prominent role of obesity-related metabolic dysregulation in the pathogenesis of these cancers. For instance, high BMI can mediate the occurrence of pancreatic cancer through C-reactive protein (CRP) as an intermediary factor (44, 45). And fat cells provide fatty acids to tumor cells, which generate energy that supports the growth and spread of cancer cells (46). Moreover, the global epidemic of non-alcoholic fatty liver disease (NAFLD) may be a notable driver of the rising burden of liver cancer, particularly in regions where obesity coexists with hepatitis virus infection (47–49). In contrast, the ASR burden of gallbladder and biliary tract cancer and leukemia showed a decline (EAPC < 0). The decline in gallbladder and biliary tract cancer may be attributed to the widespread adoption of health education and cholecystectomy (50). In contrast, the etiology of leukemia is primarily linked to environmental or genetic factors. In recent years, with economic development, increased attention has been given to environmental protection, and high BMI has been shown to have a relatively low contribution to its incidence (51). Notably, the ASMR for colon and rectal cancer and thyroid cancer remained relatively stable. The stability in colon and rectal cancer may reflect how advancements in screening technologies have partially counterbalanced obesity-associated risks (52, 53), while thyroid cancer’s relatively indolent biological characteristics likely contribute to its stable ASMR profile (54). Understanding these differences assists in formulating targeted prevention and control policies based on the prevalence patterns of major cancers. For example, for rapidly increasing cancer types such as pancreatic and liver cancers, targeted screening should be implemented in obese populations, including liver function tests combined with ultrasound surveillance for NAFLD progression, and regular imaging examinations for high-risk individuals. Additionally, public health education initiatives should be strengthened to improve awareness of the cancer risks associated with obesity.

The cancer burden attributable to high BMI exhibits notable variations across geographic regions and levels of economic development. From a geographical perspective, South Asia recorded the steepest upward trends in mortality and disability burden from 1990 to 2021, while Eastern Europe demonstrated the slowest increase. The observed disparity may reflect regional differences in the stages of obesity epidemic development, availability of healthcare resources, and effectiveness of cancer prevention policy implementation. For example, the rapidly growing cancer burden in South Asia may be linked to nutrition transition driven by accelerated urbanization, characterized by widespread adoption of high-sugar, high-fat diets and reduced physical activity, while health education and policy interventions lag (55, 56). Across socioeconomically distinct regions, notable variations exist in both the overall cancer burden and composition of cancer burden as stratified by SDI levels. High-SDI regions predominantly bear the burden of colon and rectal cancer, whereas low-SDI regions are primarily affected by liver cancer. This phenomenon may be associated with the prevalent consumption of high-fat, low-fiber, and ultra-processed foods in high-SDI countries, which elevates colon and rectal cancer risk (57). In contrast, low-SDI regions face expanded liver cancer burdens due to higher exposure to aflatoxin-contaminated foods (58), prevalent hepatitis B virus infections (59), and compounding risk factors like obesity. These findings enable context-specific adaptation of prevention and control strategies. For example, in low SDI regions, relevant institutions can be established to promote “food structural reform,” restricting the production and sale of high-calorie foods while increasing subsidies for healthy foods. Additionally, integrated interventions combining hepatitis prevention and obesity management could be implemented. For example, integrating weight monitoring into HBV patient follow-up protocols and enhancing hepatitis B vaccination coverage among populations with high BMI could be implemented.

Notably, the persistent rise in obesity-related cancer burden reflects deeper structural barriers. In high-SDI regions, marketing strategies of the food industry (such as targeted advertising of ultra-processed foods) directly contribute to the proliferation of energy-dense dietary cultures (60). In low-SDI regions, both the accessibility and affordability of healthy foods are severely constrained. Under the combined effects of poverty, imbalanced food systems, and climate change, nutrient-dense foods (fruits and vegetables) remain relatively expensive, while energy-dense but nutrient-poor processed foods become more accessible (60). This dynamic exacerbates the double burden of malnutrition, where stunting and obesity coexist. Future interventions must directly address these challenges. Policy-level interventions: Enact legislation to restrict the marketing of unhealthy food. Economic leverage: Implement fruit and vegetable subsidies in low-SDI regions.

Our analysis revealed that in 2021, women bore approximately twice the cancer-related mortality and DALY burden compared to men. The predominant cancer burden among women stems from breast and uterine cancers, likely attributable to female-specific physiological characteristics and hormonal profiles. The aromatase in adipose tissue converts androgens into estrogens, which is closely related to the development and progression of hormone-dependent breast cancer (estrogen and progesterone receptor positive) (61, 62). At the same time, women in some developing countries are prone to high BMI due to issues such as excessive nutrition during pregnancy and postpartum weight retention (63, 64), thereby increasing their risk of cancer. In contrast, the primary burden in men stems from colon and rectal cancer, liver cancer, and kidney cancer. The age distribution indicates that individuals aged 50–79 bear the major burden, which aligns with the age-related characteristics of cancer incidence (risk increases with age) (65, 66). However, the rising trend among younger populations (aged 20–24) is concerning, possibly reflecting the early impact of increasing obesity rates on cancer incidence in younger individuals. Therefore, cancer prevention and control measures should be tailored based on gender and age differences in cancer burden. First, establish gender-specific screening guidelines, such as breast cancer screening for women and liver cancer prevention for men. Second, implement a life-course weight management program, with targeted interventions during high-risk periods of weight fluctuation, such as pregnancy and menopause. Third, develop a youth obesity-cancer risk prediction model to initiate early interventions for individuals with persistent overweight.

Another innovative aspect of this study compared to previous research is the use of the ARIMA model to predict the burden of various cancers attributable to high BMI over the next decade. The burden of multiple cancers is projected to continue rising through 2031. This trend highlights the long-term carcinogenic effects of obesity as a chronic risk factor, while also being closely associated with factors such as population aging and increased consumption of processed foods (67, 68). Although the ASMR of colon and rectal cancer and uterine cancer show declining trends, their absolute burden may still increase given the persistent rise in obesity rates. These projections stress the persistent growth trend of high BMI-related cancer burden and highlight the urgency for future public health interventions. First, lifestyle interventions should be implemented for medium and low-risk populations, such as promoting low-sugar and low-fat diets to reduce calorie intake and minimize fat accumulation. Secondly, high-risk populations should undergo early cancer surveillance through standardized screening protocols (such as fecal occult blood tests, capsule endoscopy). Finally, for patients already diagnosed with cancer, a multidisciplinary team (MDT) approach should be adopted to enhance quality of life through integrated medical treatment, symptom management, psychological support, nutritional optimization, social care, and other supportive measures. However, implementing the above measures poses certain challenges. Potential challenges may include insufficient long-term public adherence, complexities in individualized implementation, and a lack of systemic and resource support. To address these challenges, we can implement tiered management strategies and promote the development of digital health tools (health monitoring apps and others).

This study also has several limitations. First, the GBD database relies on cancer registry data reported by individual countries, and statistical incompleteness due to various factors may lead to an underestimation of the disease burden. Second, during data extraction, colon cancer and rectal cancer were aggregated as “colon and rectal cancer,” while gallbladder cancer and extrahepatic bile duct cancer were combined under “gallbladder and biliary tract cancer.” These malignancies exhibit distinct variations in anatomical location, biological behavior, risk factors, and therapeutic approaches. Their aggregation may obscure significant differential trends among the constituent subtypes. This methodological limitation may compromise the accurate interpretation of disease burden dynamics and subsequently hinder the development of targeted prevention strategies. In addition, the ARIMA model does not account for unforeseen events or external shocks within its predictive framework. For instance, during pandemic lockdowns and social distancing policies, reduced outdoor activity and prolonged sedentary behavior, coupled with shifts in dietary patterns, may have exacerbated obesity rates, thereby elevating the associated disease burden (69).

## Conclusion

This study systematically evaluates the causal relationship between BMI and 12 types of cancer, along with their global burden distribution patterns, through an integrated approach combining Mendelian randomization analysis and the GBD database. This study reveals that the global burden of cancers attributable to high BMI continues to exhibit a constant growth trend. The distribution of cancer burden and the proportional contribution of specific cancers are intricately modulated by factors such as economic regions, geographic areas, sex, and age. In the future, only through multidisciplinary collaboration, precision prevention, and global governance can we effectively curb the spread of this “silent pandemic” and alleviate its long-term threats to human health.

## Data Availability

The original contributions presented in the study are included in the article/Supplementary material, further inquiries can be directed to the corresponding author.

## Footnotes

^1^ https://vizhub.healthdata.org/gbd-results/

^2^ https://gwas.mrcieu.ac.uk/
