# Supplementary material for: Global burden of cancer attributable to high BMI (1990–2031): a multidimensional analysis based on GBD and Mendelian randomization
Source: Front Nutr. 2025 Aug 5;12:1618799. doi: 10.3389/fnut.2025.1618799 (PMC12362511; doi:10.3389/fnut.2025.1618799)
Supplement: Supplementary file 1 [file Table_1.docx]

**Supplementary material**

[Figure S1 2](#_Toc12775)

[Figure S2 2](#_Toc26731)

[Figure S3 2](#_Toc18694)

[Figure S4 3](#_Toc3690)

[Table S1 5](#_Toc28049)

[Table S2 7](#_Toc21332)

[Table S3 9](#_Toc9428)

[Table S4 10](#_Toc17071)

[Table S5. 12](#_Toc8700)

Figure S1 Temporal trends in the proportional distribution of cancer types attributable to high BMI across global and different socioeconomic regions, 1990-2021 ( Mortality).


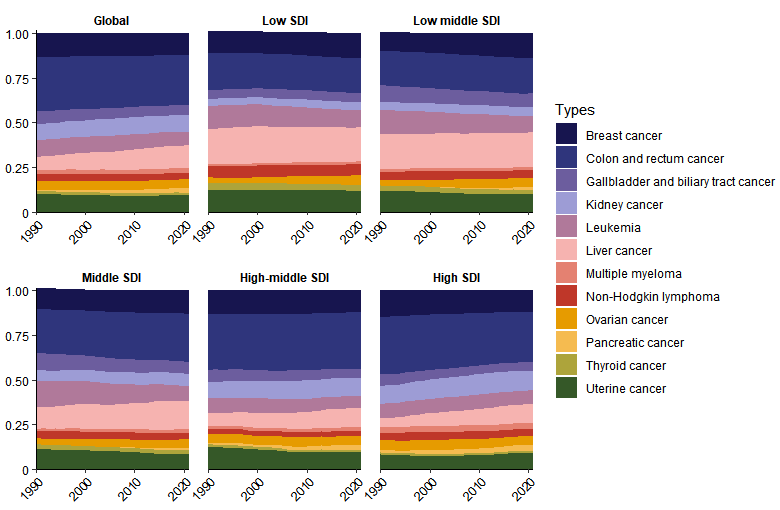


Figure S2 Temporal trends in cancer mortality attributable to high BMI across global and different socioeconomic regions, 1990–2021

**
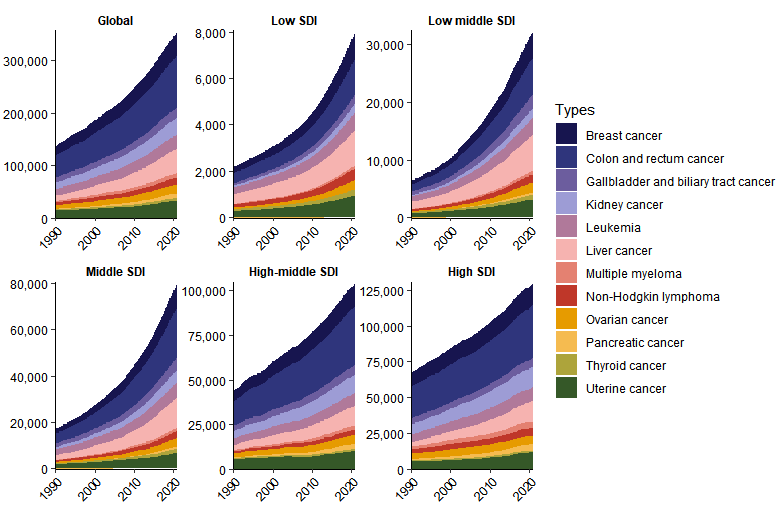
**

Figure S3 Temporal trends in cancer DALYs attributable to high BMI across global and different socioeconomic regions, 1990–2021

**
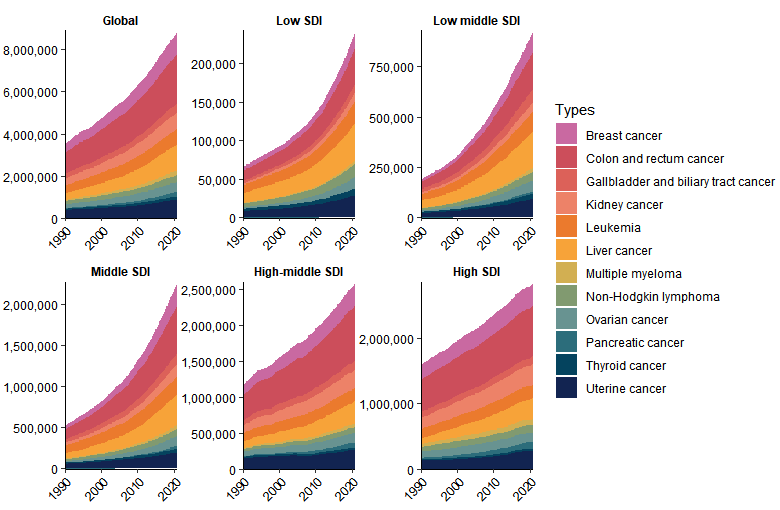
**

Figure S4 The trend in age-standardized mortality (a) and DALYs (b) rates of cancer attributable to high BMI in 21 GBD regions by SDI, 1990–2021.


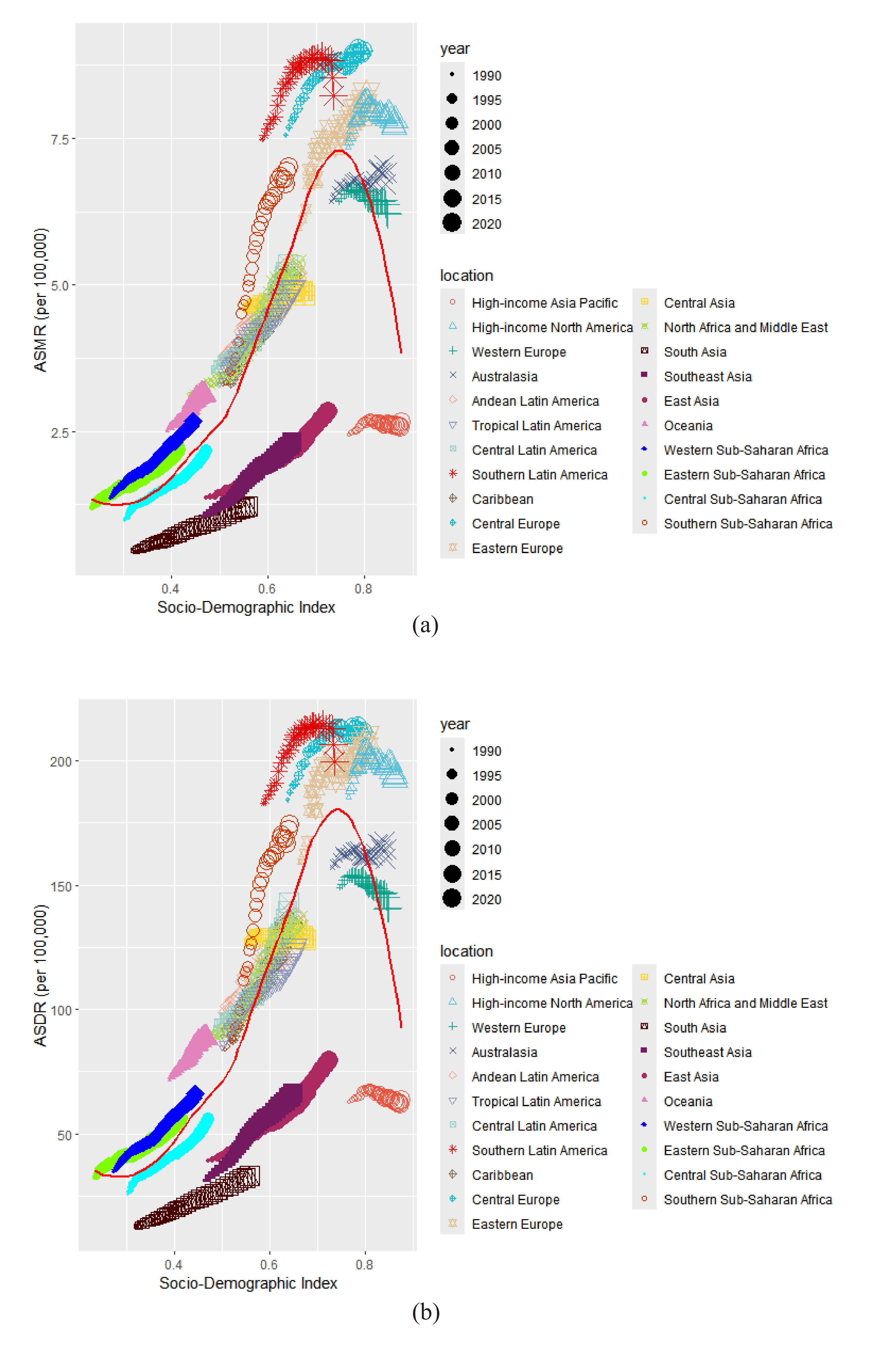


Table S1 Mortality Cases and ASMR of cancer attributable to high BMI in 1990 and 2021, and EAPC of ASMR From 1990 to 2021. ( In the Table, red indicates the maximum or top two EAPC values within each subgroup, while blue represents the minimum or bottom two EAPC values )

|  | **1990** | | **2021** | | **1990-2021** |
| --- | --- | --- | --- | --- | --- |
| **Characteristics** | **Mortality cases**  **(95% UI)** | **ASMR per 100,000**  **(95% UI)** | **Mortality cases**  **(95% UI)** | **ASMR per 100,000**  **(95% UI)** | **EAPC of ASMR**  **(95% UI)** |
| Global | 137,353 (57,450 to 225,297) | 3.66 (1.51 to 6.03) | 356,738 (146,116 to 581,012) | 4.18 (1.71 to 6.8) | 0.35 (0.32,0.38) |
| **Sex** | | | | | |
| Male | 46,512 (21,649 to 74,729) | 2.73 (1.27 to 4.39) | 139,860 (61,467 to 227,901) | 3.6 (1.58 to 5.85) | - |
| Female | 90,841 (36,243 to 148,645) | 4.38 (1.73 to 7.18) | 216,879 (84,421 to 357,832) | 4.67 (1.83 to 7.69) | - |
| **SDI region** | | | | | |
| Low | 2,150 (1,001 to 3,336) | 0.94 (0.42 to 1.46) | 8,042 (3,472 to 12,804) | 1.59 (0.65 to 2.56) | 1.65 (1.55,1.74) |
| Low-middle | 6,380 (3,153 to 9,772) | 1.06 (0.51 to 1.63) | 32,289 (13,924 to 51,826) | 2.25 (0.96 to 3.62) | 2.63 (2.57,2.69) |
| Middle | 16,998 (8,408 to 27,188) | 1.65 (0.8 to 2.66) | 80,557 (34,322 to 131,379) | 2.99 (1.28 to 4.88) | 1.91 (1.88,1.94) |
| High-middle | 44,038 (18,883 to 72,250) | 4.51 (1.93 to 7.41) | 104,828 (42,727 to 171,537) | 5.3 (2.17 to 8.66) | 0.4 (0.34,0.47) |
| High | 67,534 (26,500 to 111,772) | 6.08 (2.39 to 10.07) | 130,515 (51,156 to 215,588) | 6.04 (2.39 to 9.94) | -0.11 (-0.15,-0.06) |
| **Causes** | | | | | |
| Breast cancer | 18,745 (-604 to 38,157) | 0.52 (-0.02 to 1.06) | 44,707 (-1,478 to 89,575) | 0.52 (-0.02 to 1.03) | -0.09 (-0.13,-0.05) |
| Colon and rectum cancer | 41,536 (17,666 to 67,379) | 1.14 (0.48 to 1.86) | 99,268 (42,956 to 157,949) | 1.17 (0.51 to 1.87) | 0 (-0.04,0.04) |
| Gallbladder and biliary tract cancer | 9,680 (6,644 to 13,261) | 0.26 (0.18 to 0.36) | 20,102 (13,656 to 27,792) | 0.24 (0.16 to 0.33) | -0.4 (-0.5,-0.3) |
| Kidney cancer | 12,112 (4,755 to 19,780) | 0.32 (0.12 to 0.52) | 32,363 (13,159 to 52,691) | 0.38 (0.15 to 0.62) | 0.54 (0.46,0.63) |
| Leukemia | 12,995 (9,657 to 16,777) | 0.33 (0.25 to 0.43) | 27,014 (19,712 to 34,956) | 0.32 (0.23 to 0.42) | -0.12 (-0.18,-0.06) |
| Liver cancer | 10,282 (4,197 to 16,722) | 0.26 (0.11 to 0.42) | 46,204 (18,606 to 77,983) | 0.53 (0.21 to 0.9) | 2.36 (2.27,2.44) |
| Multiple myeloma | 3,023 (-1,047 to 7,783) | 0.08 (-0.03 to 0.21) | 9,165 (-3,675 to 22,992) | 0.11 (-0.04 to 0.27) | 0.9 (0.74,1.05) |
| Non-Hodgkin lymphoma | 5,083 (1,724 to 8,609) | 0.13 (0.04 to 0.22) | 12,966 (4,319 to 22,308) | 0.15 (0.05 to 0.26) | 0.18 (0.08,0.29) |
| Ovarian cancer | 6,850 (1,423 to 12,865) | 0.18 (0.04 to 0.33) | 17,344 (4,141 to 30,810) | 0.2 (0.05 to 0.36) | 0.32 (0.22,0.42) |
| Pancreatic cancer | 955 (-2,011 to 5,926) | 0.02 (-0.05 to 0.16) | 9,215 (-2,377 to 26,607) | 0.11 (-0.03 to 0.31) | 4.77 (4.41,5.14) |
| Thyroid cancer | 2,198 (1,642 to 2,818) | 0.06 (0.04 to 0.07) | 5,255 (3,914 to 6,653) | 0.06 (0.05 to 0.08) | 0 (0,0) |
| Uterine cancer | 13,893 (9,874 to 18,653) | 0.37 (0.26 to 0.49) | 33,134 (23,878 to 43,299) | 0.39 (0.28 to 0.5) | 0.06 (-0.05,0.17) |
| **Region** | | | | | |
| Andean Latin America | 714 (373 to 1,129) | 3.47 (1.77 to 5.49) | 3,068 (1,459 to 5,088) | 5.2 (2.46 to 8.64) | 1.26 (1.18,1.34) |
| Australasia | 1,496 (559 to 2,528) | - | 3,822 (1,473 to 6,370) | - | 0.17 (0.12,0.22) |
| Caribbean | 851 (361 to 1,366) | 3.32 (1.38 to 5.33) | 2,801 (1,200 to 4,597) | 5.17 (2.23 to 8.48) | 1.48 (1.43,1.53) |
| Central Asia | 2,194 (965 to 3,534) | 4.61 (2.03 to 7.44) | 3,992 (1,691 to 6,589) | 4.83 (2.04 to 8) | 0.24 (0.17,0.3) |
| Central Europe | 11,259 (4,863 to 18,159) | 7.56 (3.27 to 12.18) | 20,488 (8,452 to 33,970) | 9 (3.72 to 14.91) | 0.47 (0.39,0.56) |
| Central Latin America | 2,903 (1,392 to 4,613) | 3.53 (1.65 to 5.65) | 13,461 (5,928 to 22,110) | 5.36 (2.36 to 8.83) | 1.29 (1.17,1.4) |
| Central Sub-Saharan  Africa | 223 (84 to 367) | 0.99 (0.37 to 1.64) | 1,166 (417 to 2,078) | 2.17 (0.74 to 3.94) | 2.5 (2.38,2.62) |
| East Asia | 12,006 (6,255 to 18,964) | 1.36 (0.68 to 2.16) | 61,372 (25,647 to 104,532) | 2.84 (1.21 to 4.81) | 2.39 (2.34,2.44) |
| Eastern Europe | 17,001 (7,179 to 27,102) | 1.18 (0.54 to 1.86) | 29,386 (11,873 to 48,273) | - | 0.83 (0.72,0.94) |
| Eastern Sub-Saharan Africa | 893 (424 to 1,386) | - | 3,607 (1,440 to 5,938) | 2.18 (0.83 to 3.63) | 1.89 (1.81,1.97) |
| High-income Asia Pacific | 4,858 (2,569 to 7,512) | - | 13,084 (5,858 to 20,819) | - | 0.06 (-0.03,0.15) |
| High-income North America | 25,893 (9,553 to 43,317) | 7.35 (2.71 to 12.28) | 52,033 (20,448 to 85,882) | 7.8 (3.1 to 12.83) | 0.07 (-0.02,0.17) |
| North Africa and Mid dle East | 5,221 (2,494 to 8,137) | 3.13 (1.48 to 4.9) | 23,639 (10,326 to 37,939) | 5.35 (2.29 to 8.66) | 1.94 (1.86,2.01) |
| Oceania | 77 (32 to 127) | 2.5 (1.02 to 4.14) | 248 (97 to 411) | 3.13 (1.21 to 5.17) | 0.73 (0.71,0.76) |
| South Asia | 2,726 (1,479 to 4,142) | 0.47 (0.25 to 0.71) | 18,111 (8,235 to 28,683) | 1.22 (0.55 to 1.93) | 3.21 (3.18,3.24) |
| Southeast Asia | 2,841 (1,416 to 4,443) | 1.04 (0.51 to 1.63) | 15,689 (6,553 to 25,864) | 2.31 (0.97 to 3.81) | 2.59 (2.47,2.71) |
| Southern Latin America | 3,425 (1,445 to 5,664) | 7.49 (3.15 to 12.42) | 7,242 (2,956 to 11,817) | 8.23 (3.37 to 13.37) | 0.45 (0.32,0.58) |
| Southern Sub-Saharan Africa | 862 (324 to 1,401) | 3.29 (1.2 to 5.4) | 3,886 (1,393 to 6,487) | 7.01 (2.48 to 11.78) | 2.56 (2.26,2.86) |
| Tropical Latin America | 2,958 (1,303 to 4,828) | 3.31 (1.42 to 5.42) | 12,686 (5,023 to 21,003) | 4.95 (1.96 to 8.2) | 1.24 (1.18,1.29) |
| Western Europe | 37,785 (14,455 to 63,503) | - | 61,942 (22,695 to 105,818) | - | -0.14 (-0.18,-0.1) |
| Western Sub-Saharan Africa | 1,170 (387 to 1,963) | 1.37 (0.43 to 2.32) | 5,016 (1,461 to 8,666) | 2.68 (0.76 to 4.61) | 2.13 (2.06,2.2) |

**Table S2** DALYs and ASDR of cancer attributable to high BMI in 1990 and 2021, and EAPC of ASDR From 1990 to 2021. ( In the Table, red indicates the maximum or top two EAPC values within each subgroup, while blue represents the minimum or bottom two EAPC values )

|  | **1990** | | **2021** | | **1990-2021** |
| --- | --- | --- | --- | --- | --- |
| **Characteristics** | **DALYs cases**  **(95% UI)** | **ASDR per 100,000**  **(95% UI)** | **DALYs cases**  **(95% UI)** | **ASDR per 100,000**  **(95% UI)** | **EAPC of ASDR**  **(95% UI)** |
| Global | 3,549,049 (1,548,429 to 5,731,481) | 87.53 (37.43 to 141.83) | 8,894,525 (3,751,953 to 14,385,271) | 102.17 (43.24 to 165.02) | 0.42 (0.39,0.46) |
| **Sex** | | | | | |
| Male | 1,290,002 (618,507 to 2,058,112) | 66.66 (31.39 to 106.64) | 3,628,748 (1,617,744 to 5,913,529) | 88.18 (39.36 to 143.4) | - |
| Female | 2,259,047 (941,000 to 3,670,501) | 105.26 (43.35 to 171.59) | 5,265,777 (2,095,175 to 8,559,032) | 114.61 (46.08 to 185.86) | - |
| **SDI region** | | | | | |
| Low | 65,396 (32,498 to 99,595) | 25.53 (12 to 39.52) | 242,590 (112,346 to 379,286) | 42.24 (18.36 to 67.28) | 1.54 (1.45,1.63) |
| Low-middle | 191,455 (101,463 to 287,711) | 28.1 (14.16 to 42.84) | 927,919 (417,853 to 1,467,815) | 59.9 (26.35 to 95.37) | 2.64 (2.57,2.70) |
| Middle | 524,985 (272,944 to 820,270) | 44.73 (22.44 to 70.85) | 2,270,687 (989,854 to 3,669,207) | 80.52 (35.34 to 129.76) | 1.89 (1.86,1.92) |
| High-middle | 1,174,635 (516,167 to 1,903,228) | 114.13 (50.1 to 184.97) | 2,594,673 (1,071,558 to 4,227,618) | 132.3 (55.35 to 215.07) | 0.33 (0.27,0.39) |
| High | 1,586,175 (632,597 to 2,600,946) | 146.13 (58.5 to 239.35) | 2,846,739 (1,138,384 to 4,669,847) | 146.22 (59.75 to 237.64) | -0.06 (-0.10,-0.02) |
| **Causes** |  |  |  |  |  |
| Breast cancer | 429,066 (-20,024 to 868,901) | 11.19 (-0.41 to 22.71) | 1,041,309 (-40,216 to 2,029,537) | 11.63 (-0.49 to 22.68) | 0.04 (0,0.08) |
| Colon and rectum cancer | 1,015,042 (429,787 to 1,631,974) | 25.54 (10.83 to 41.2) | 2,364,664 (1,021,594 to 3,752,340) | 27.33 (11.8 to 43.37) | 0.12 (0.08,0.16) |
| Gallbladder and biliary tract cancer | 227,609 (156,891 to 309,773) | 5.74 (3.95 to 7.83) | 451,489 (308,188 to 621,591) | 5.2 (3.56 to 7.17) | -0.44 (-0.52,-0.37) |
| Kidney cancer | 318,070 (125,099 to 517,060) | 7.8 (3.06 to 12.69) | 781,627 (319,389 to 1,260,313) | 8.99 (3.68 to 14.51) | 0.41 (0.32,0.5) |
| Leukemia | 404,036 (300,964 to 520,064) | 9.22 (6.86 to 11.9) | 741,191 (557,919 to 955,338) | 8.72 (6.56 to 11.24) | -0.23 (-0.27,-0.18) |
| Liver cancer | 292,696 (119,095 to 475,963) | 6.97 (2.84 to 11.34) | 1,237,313 (504,239 to 2,101,958) | 14.16 (5.77 to 24.06) | 2.31 (2.22,2.39) |
| Multiple myeloma | 72,010 (-25,461 to 184,167) | 1.81 (-0.64 to 4.65) | 207,634 (-84,340 to 515,477) | 2.39 (-0.97 to 5.94) | 0.76 (0.67,0.86) |
| Non-Hodgkin lymphoma | 145,238 (49,395 to 245,905) | 3.45 (1.17 to 5.85) | 343,580 (114,574 to 593,411) | 4 (1.33 to 6.9) | 0.21 (0.11,0.32) |
| Ovarian cancer | 188,874 (38,401 to 355,691) | 4.57 (0.94 to 8.6) | 477,248 (113,449 to 840,002) | 5.46 (1.3 to 9.62) | 0.47 (0.42,0.52) |
| Pancreatic cancer | 21,952 (-49,859 to 141,075) | 0.55 (-1.24 to 3.53) | 223,368 (-47,782 to 626,550) | 2.54 (-0.56 to 7.16) | 4.85 (4.6,5.1) |
| Thyroid cancer | 61,815 (46,571 to 79,116) | 1.49 (1.12 to 1.9) | 144,955 (109,230 to 184,747) | 1.68 (1.26 to 2.14) | 0.38 (0.35,0.42) |
| Uterine cancer | 372,641 (264,224 to 500,197) | 9.19 (6.52 to 12.32) | 880,147 (631,165 to 1160,930) | 10.07 (7.22 to 13.28) | 0.22 (0.12,0.32) |
| **Region** | | | | | |
| Andean Latin America | 20,888 (11,342 to 32,509) | 93.31 (49.3 to 146.82) | 82,761 (40,705 to 135,222) | 135.71 (66.24 to 222.18) | 1.15 (1.06,1.23) |
| Australasia | 36,211 (13,825 to 60,381) | 157.23 (59.8 to 262.1) | 82,959 (32,325 to 137,498) | 164.54 (65.3 to 270.81) | 0.07 (0.03,0.12) |
| Caribbean | 23,434 (10,740 to 36,678) | 87.48 (38.78 to 137.94) | 72,468 (32,307 to 117,854) | 134.39 (60.55 to 218.18) | 1.43 (1.37,1.48) |
| Central Asia | 63,636 (29,265 to 101,458) | 126.53 (58.1 to 201.66) | 114,780 (50,509 to 187,073) | 127.99 (56.04 to 208.87) | 0.05 (0,0.1) |
| Central Europe | 281,016 (122,808 to 450,030) | 184.28 (81.08 to 294.68) | 452,919 (189,278 to 747,241) | 211.7 (89.16 to 348.4) | 0.37 (0.28,0.47) |
| Central Latin America | 83,167 (42,004 to 129,673) | 91.16 (44.07 to 144.34) | 370,265 (168,146 to 594,003) | 142.9 (64.74 to 229.64) | 1.38 (1.26,1.5) |
| Central Sub-Saharan  Africa | 6,597 (2,592 to 10,752) | - | 34,810 (13,126 to 61,595) | - | 2.43 (2.32,2.54) |
| East Asia | 391,398 (210,762 to 609,910) | 39.11 (20.44 to 61.38) | 1,724,414 (722,814 to 2,948,904) | 79.51 (34.05 to 134.88) | 2.31 (2.25,2.37) |
| Eastern Europe | 460,086 (197,435 to 732,942) | 159.54 (69.63 to 251.23) | 725,101 (295,196 to 1,181,739) | 209.44 (86.18 to 340.07) | 0.62 (0.49,0.75) |
| Eastern Sub-Saharan Africa | 27,283 (14,004 to 40,664) | - | 107,868 (46,042 to 174,842) | - | 1.7 (1.62,1.78) |
| High-income Asia Pacific | 129,249 (67,178 to 201,227) | 62.81 (32.83 to 97.49) | 255,553 (109,446 to 408,426) | 63.43 (26.94 to 100.87) | -0.17 (-0.25,-0.08) |
| High-income North America | 622,230 (235,189 to 1,029,764) | 185.35 (70.01 to 307.11) | 1,207,599 (490,749 to 1,967,531) | 194.14 (80.69 to 313.02) | 0.05 (-0.04,0.14) |
| North Africa and Mid dle East | 157,548 (77,816 to 243,987) | 82.92 (39.92 to 129.17) | 672,534 (306,994 to 1,059,584) | 135.85 (60.05 to 216.87) | 1.76 (1.67,1.85) |
| Oceania | 2,576 (1,088 to 4,215) | 71.44 (29.64 to 117.8) | 8,105 (3,238 to 13,360) | 88.56 (34.75 to 146.19) | 0.69 (0.66,0.72) |
| South Asia | 84,091 (49,190 to 124,725) | 12.78 (7 to 19.3) | 521,409 (250,127 to 802,843) | 32.86 (15.34 to 51.13) | 3.17 (3.14,3.21) |
| Southeast Asia | 95,314 (49,484 to 147,277) | 31.15 (15.66 to 48.51) | 483,637 (203,365 to 794,583) | 66.15 (28.04 to 108.67) | 2.43 (2.29,2.57) |
| Southern Latin America | 85,657 (37,444 to 140,449) | 183.03 (79.82 to 300.11) | 170,041 (71,090 to 272,894) | 199.53 (83.98 to 319.44) | 0.43 (0.31,0.55) |
| Southern Sub-Saharan Africa | 24,512 (9,753 to 39,136) | - | 107,576 (39,978 to 176,891) | - | 2.52 (2.22,2.82) |
| Tropical Latin America | 83,167 (38,949 to 133,345) | 84.62 (37.85 to 137.74) | 328,005 (134,611 to 529,825) | 125.23 (51.52 to 202.39) | 1.15 (1.1,1.21) |
| Western Europe | 838,339 (322,841 to 1,406,793) | 149.11 (58.17 to 249.01) | 1,230,583 (457,928 to 2,086,858) | 140.74 (53.72 to 237.26) | -0.21 (-0.26,-0.16) |
| Western Sub-Saharan Africa | 32,649 (11,561 to 53,435) | - | 141,136 (44,029 to 241,543) | - | 2.01 (1.93,2.09) |

Table S3. The actual and projected values of ASMR among six cancer types from 1990 to 2031, stratified by cancer types.

| **Year** | **Actual or Forecast** | **ASMR** | | | | | |
| --- | --- | --- | --- | --- | --- | --- | --- |
|  |  | **Breast cancer** | **Colon and rectum cancer** | **Gallbladder and biliary tract cancer** | **Kidney cancer** | **Leukemia** | **Liver cancer** |
| 1990 | Actual | 0.52 | 1.14 | 0.26 | 0.32 | 0.33 | 0.26 |
| 1991 | Actual | 0.52 | 1.14 | 0.26 | 0.32 | 0.33 | 0.27 |
| 1992 | Actual | 0.52 | 1.15 | 0.26 | 0.33 | 0.33 | 0.27 |
| 1993 | Actual | 0.53 | 1.16 | 0.26 | 0.34 | 0.33 | 0.28 |
| 1994 | Actual | 0.53 | 1.17 | 0.26 | 0.34 | 0.34 | 0.29 |
| 1995 | Actual | 0.53 | 1.17 | 0.26 | 0.35 | 0.34 | 0.3 |
| 1996 | Actual | 0.53 | 1.17 | 0.25 | 0.34 | 0.34 | 0.31 |
| 1997 | Actual | 0.53 | 1.16 | 0.25 | 0.35 | 0.33 | 0.32 |
| 1998 | Actual | 0.53 | 1.17 | 0.25 | 0.35 | 0.34 | 0.33 |
| 1999 | Actual | 0.53 | 1.17 | 0.25 | 0.35 | 0.34 | 0.34 |
| 2000 | Actual | 0.53 | 1.18 | 0.25 | 0.36 | 0.34 | 0.35 |
| 2001 | Actual | 0.53 | 1.18 | 0.25 | 0.36 | 0.34 | 0.36 |
| 2002 | Actual | 0.53 | 1.18 | 0.24 | 0.36 | 0.34 | 0.37 |
| 2003 | Actual | 0.53 | 1.18 | 0.24 | 0.37 | 0.34 | 0.37 |
| 2004 | Actual | 0.53 | 1.17 | 0.24 | 0.37 | 0.34 | 0.37 |
| 2005 | Actual | 0.52 | 1.17 | 0.24 | 0.37 | 0.34 | 0.38 |
| 2006 | Actual | 0.52 | 1.16 | 0.24 | 0.37 | 0.34 | 0.39 |
| 2007 | Actual | 0.52 | 1.15 | 0.24 | 0.37 | 0.33 | 0.4 |
| 2008 | Actual | 0.52 | 1.16 | 0.23 | 0.38 | 0.33 | 0.41 |
| 2009 | Actual | 0.52 | 1.15 | 0.23 | 0.38 | 0.33 | 0.42 |
| 2010 | Actual | 0.52 | 1.15 | 0.23 | 0.38 | 0.33 | 0.43 |
| 2011 | Actual | 0.52 | 1.15 | 0.23 | 0.38 | 0.33 | 0.44 |
| 2012 | Actual | 0.51 | 1.15 | 0.23 | 0.38 | 0.33 | 0.45 |
| 2013 | Actual | 0.51 | 1.15 | 0.23 | 0.38 | 0.33 | 0.46 |
| 2014 | Actual | 0.51 | 1.15 | 0.23 | 0.38 | 0.33 | 0.48 |
| 2015 | Actual | 0.51 | 1.15 | 0.23 | 0.39 | 0.33 | 0.49 |
| 2016 | Actual | 0.52 | 1.16 | 0.23 | 0.39 | 0.33 | 0.5 |
| 2017 | Actual | 0.52 | 1.16 | 0.23 | 0.38 | 0.33 | 0.5 |
| 2018 | Actual | 0.52 | 1.17 | 0.24 | 0.38 | 0.33 | 0.51 |
| 2019 | Actual | 0.52 | 1.17 | 0.24 | 0.38 | 0.32 | 0.52 |
| 2020 | Actual | 0.51 | 1.17 | 0.24 | 0.38 | 0.32 | 0.52 |
| 2021 | Actual | 0.52 | 1.17 | 0.24 | 0.38 | 0.32 | 0.53 |
| 2022 | Forecast | 0.52 | 1.168623 | 0.24 | 0.3819355 | 0.32 | 0.5387097 |
| 2023 | Forecast | 0.52 | 1.167435 | 0.24 | 0.383871 | 0.32 | 0.5474194 |
| 2024 | Forecast | 0.52 | 1.166409 | 0.24 | 0.3858065 | 0.32 | 0.556129 |
| 2025 | Forecast | 0.52 | 1.165523 | 0.24 | 0.3877419 | 0.32 | 0.5648387 |
| 2026 | Forecast | 0.52 | 1.164759 | 0.24 | 0.3896774 | 0.32 | 0.5735484 |
| 2027 | Forecast | 0.52 | 1.164099 | 0.24 | 0.3916129 | 0.32 | 0.5822581 |
| 2028 | Forecast | 0.52 | 1.163529 | 0.24 | 0.3935484 | 0.32 | 0.5909677 |
| 2029 | Forecast | 0.52 | 1.163037 | 0.24 | 0.3954839 | 0.32 | 0.5996774 |
| 2030 | Forecast | 0.52 | 1.162612 | 0.24 | 0.3974194 | 0.32 | 0.6083871 |
| 2031 | Forecast | 0.52 | 1.162246 | 0.24 | 0.3993548 | 0.32 | 0.6170968 |

Table S4. The actual and projected values of ASMR among the remaining six cancer types from 1990 to 2031, stratified by cancer types.

| **Year** | **Actual or Forecast** | **ASMR** | | | | | |
| --- | --- | --- | --- | --- | --- | --- | --- |
|  |  | **Multiple myeloma** | **Non-Hodgkin lymphoma** | **Ovarian cancer** | **Pancreatic cancer** | **Thyroid cancer** | **Uterine cancer** |
| 1990 | Actual | 0.08 | 0.13 | 0.18 | 0.02 | 0.06 | 0.37 |
| 1991 | Actual | 0.08 | 0.14 | 0.18 | 0.03 | 0.06 | 0.37 |
| 1992 | Actual | 0.08 | 0.14 | 0.18 | 0.03 | 0.06 | 0.37 |
| 1993 | Actual | 0.09 | 0.14 | 0.18 | 0.03 | 0.06 | 0.37 |
| 1994 | Actual | 0.09 | 0.15 | 0.18 | 0.03 | 0.06 | 0.37 |
| 1995 | Actual | 0.09 | 0.15 | 0.19 | 0.04 | 0.06 | 0.37 |
| 1996 | Actual | 0.09 | 0.15 | 0.19 | 0.04 | 0.06 | 0.37 |
| 1997 | Actual | 0.09 | 0.15 | 0.19 | 0.04 | 0.06 | 0.36 |
| 1998 | Actual | 0.09 | 0.15 | 0.19 | 0.04 | 0.06 | 0.36 |
| 1999 | Actual | 0.1 | 0.15 | 0.19 | 0.04 | 0.06 | 0.36 |
| 2000 | Actual | 0.1 | 0.15 | 0.19 | 0.05 | 0.06 | 0.36 |
| 2001 | Actual | 0.1 | 0.15 | 0.19 | 0.05 | 0.06 | 0.36 |
| 2002 | Actual | 0.1 | 0.15 | 0.2 | 0.05 | 0.06 | 0.36 |
| 2003 | Actual | 0.1 | 0.15 | 0.2 | 0.05 | 0.06 | 0.37 |
| 2004 | Actual | 0.1 | 0.15 | 0.2 | 0.06 | 0.06 | 0.36 |
| 2005 | Actual | 0.1 | 0.15 | 0.2 | 0.06 | 0.06 | 0.36 |
| 2006 | Actual | 0.1 | 0.15 | 0.2 | 0.06 | 0.06 | 0.35 |
| 2007 | Actual | 0.1 | 0.15 | 0.2 | 0.07 | 0.06 | 0.35 |
| 2008 | Actual | 0.1 | 0.15 | 0.2 | 0.07 | 0.06 | 0.35 |
| 2009 | Actual | 0.1 | 0.15 | 0.2 | 0.07 | 0.06 | 0.35 |
| 2010 | Actual | 0.1 | 0.15 | 0.2 | 0.07 | 0.06 | 0.35 |
| 2011 | Actual | 0.1 | 0.15 | 0.2 | 0.08 | 0.06 | 0.35 |
| 2012 | Actual | 0.1 | 0.15 | 0.19 | 0.08 | 0.06 | 0.36 |
| 2013 | Actual | 0.1 | 0.15 | 0.19 | 0.08 | 0.06 | 0.36 |
| 2014 | Actual | 0.1 | 0.15 | 0.2 | 0.08 | 0.06 | 0.36 |
| 2015 | Actual | 0.11 | 0.15 | 0.19 | 0.09 | 0.06 | 0.37 |
| 2016 | Actual | 0.11 | 0.15 | 0.2 | 0.09 | 0.06 | 0.37 |
| 2017 | Actual | 0.11 | 0.15 | 0.2 | 0.1 | 0.06 | 0.38 |
| 2018 | Actual | 0.11 | 0.15 | 0.2 | 0.1 | 0.06 | 0.38 |
| 2019 | Actual | 0.11 | 0.15 | 0.2 | 0.1 | 0.06 | 0.38 |
| 2020 | Actual | 0.11 | 0.15 | 0.2 | 0.1 | 0.06 | 0.38 |
| 2021 | Actual | 0.11 | 0.15 | 0.2 | 0.11 | 0.06 | 0.39 |
| 2022 | Forecast | 0.1109677 | 0.15 | 0.1999901 | 0.1084664 | 0.06 | 0.3884006 |
| 2023 | Forecast | 0.1119355 | 0.15 | 0.1999901 | 0.111158 | 0.06 | 0.3869353 |
| 2024 | Forecast | 0.1129032 | 0.15 | 0.1999901 | 0.1138496 | 0.06 | 0.3855928 |
| 2025 | Forecast | 0.113871 | 0.15 | 0.1999901 | 0.1165412 | 0.06 | 0.3843628 |
| 2026 | Forecast | 0.1148387 | 0.15 | 0.1999901 | 0.1192327 | 0.06 | 0.383236 |
| 2027 | Forecast | 0.1158065 | 0.15 | 0.1999901 | 0.1219243 | 0.06 | 0.3822037 |
| 2028 | Forecast | 0.1167742 | 0.15 | 0.1999901 | 0.1246159 | 0.06 | 0.3812579 |
| 2029 | Forecast | 0.1177419 | 0.15 | 0.1999901 | 0.1273075 | 0.06 | 0.3803913 |
| 2030 | Forecast | 0.1187097 | 0.15 | 0.1999901 | 0.1299991 | 0.06 | 0.3795975 |
| 2031 | Forecast | 0.1196774 | 0.15 | 0.1999901 | 0.1326907 | 0.06 | 0.3788702 |

Table S5. Information of the exposures and outcome datasets.

| **IEU GWAS id** | **Exposure or outcome** | **Identified SNPs** |
| --- | --- | --- |
| ukb-b-2303 | Body mass index (BMI) | 448 |
| ukb-d-D12 | Benign neoplasm of colon,rectum, anus and anal canal | 374 |
| ieu-b-4915 | Liver & bile duct cancer | 335 |
| ebi-a-GCST90018893 | Pancreatic cancer | 372 |
| ukb-a-519 | Malignant neoplasm of breast | 372 |
| ukb-b-13545 | uterine/endometrial cancer | 203 |
| ieu-b-4963 | Ovarian cancer | 345 |
| finn-b-C3_KIDNEY_NOTRENALPELVIS | Malignant neoplasm of kidney | 350 |
| ebi-a-GCST90018929 | Thyroid cancer | 372 |
| ebi-a-GCST90018878 | Malignant lymphoma | 372 |
| ieu-b-4957 | Multiple myeloma | 343 |
| finn-b-CD2_LEUKAEMIA_NAS | Leukaemia of unspecified cell type | 350 |

The information of the exposure and outcome datasets. SNPs, Single-nucleotide polymorphisms. IEU, Integrative Epidemiology Unit; GWAS, Genome-Wide Association Studies
